# The Feasibility of Exosome-Enriched Platelet-rich Fibrin (PRF) for the Treatment of Gingival Recessions: A Case Series of 27 Patients

**DOI:** 10.3290/j.ohpd.c_2394

**Published:** 2026-01-28

**Authors:** Scott Froum, Nathan E. Estrin, Paras Ahmad, Nima Farshidfar, Richard J. Miron

**Affiliations:** a Scott Froum Professor, Department of Periodontology, School of Dental Medicine, Stony Brook University, Stony Brook, NY, USA. Conceptualization, methodology, formal analysis, investigation, prepared original draft, reviewed and edited the manuscript, supervised the project, project administration.; b Nathan Estrin Professor, The University of Iowa College of Dentistry and Dental Clinics, Iowa City, IA, USA. Conceptualization, methodology, formal analysis, investigation, resources, prepared original draft, reviewed and edited the manuscript, project administration.; c Paras Ahmad Post-doctoral Student, Department of Research, The Miron Lab, Jupiter, FL, USA. Methodology, resources, prepared original draft, reviewed and edited the manuscript.; d Nima Farshidfar PhD Student, Department of Periodontology, University of Bern, Bern, Switzerland. Methodology, resources, prepared original draft, reviewed and edited the manuscript.; e Richard J. Miron Professor, Department of Research, The Miron Lab, Jupiter, FL, USA; Department of Periodontology, University of Bern, Bern, Switzerland. Conceptualization, methodology, investigation, prepared original draft, reviewed and edited the manuscript, supervised the project. All authors read and agreed to the published version of the manuscript.

**Keywords:** extracellular vesicles, gingiva, recession, regeneration, platelet-rich fibrin, PRF.

## Abstract

**Purpose:**

Given the emerging potential of bioactive, cell-free regenerative therapies, integrating exosomes into dental practice may provide a biologically driven, minimally invasive method to improve soft tissue healing and root coverage outcomes. Hence, this first case series aimed to clinically assess the efficacy and safety of exosome-enriched solid platelet-rich fibrin (Exos-solid-PRF) to treat gingival recessions.

**Materials and Methods:**

Twenty-seven patients (125 teeth) treated between January 2023 and July 2024 using a minimally invasive vestibular access technique with Exos-solid-PRF and liquid-PRF were included. Autologous PRF was prepared via horizontal centrifugation and hydrated with exosomes before surgical application. Clinical parameters, such as attachment gain (AG), keratinized tissue (KT), recession depth (RD), and recession width (RW), were measured at baseline and at a 6-month follow-up.

**Results:**

According to the Cairo classification system for recession, out of 125 teeth, 23 (18.4%) were recession type 1 (RT1) and 102 (81.6%) were RT2. Statistically significant improvements were observed in AG (+0.46 ± 0.84mm), RD (-1.93 ± 1.05 mm), and RW (-1.43 ± 1.43mm), with a mean root coverage of 68% and complete root coverage in 34 teeth. RT1 cases demonstrated 86% coverage, and RT2 cases achieved 64%, exhibiting the regenerative potential of this novel therapy in treating both isolated and multiple adjacent gingival recessions, particularly in the esthetic zone.

**Conclusions:**

While the outcomes for RT1 defects are comparable to those of gold-standard CTG approaches, the modest improvements in RT2 defects reflect both the potential and the limitations of this novel method. Additional controlled studies, long-term follow-up, and mechanistic investigations are required to validate these outcomes and optimize the therapeutic application of exosomes in clinical periodontology.

Tissue engineering requires a complex interplay between signaling molecules, growth factors, and native cells, together with a matrix, which acts as a framework for regeneration.^53^ The basis of tissue engineering lies in triggering a sequential cascade of events that facilitates the organized migration of cells from surrounding tissues into the defect.^31^ One of the obstacles of both soft and hard tissue regeneration is providing a bioactive matrix to serve as a framework for cell migration while also capable of releasing specific signaling molecules and growth factors.^31^ The ideal scaffold functions as a framework for growth factors and extracellular cells while also acting as a matrix that promotes and modulates cellular processes including migration, cell synthesis, and mitosis.^54^

One of the main aims of periodontal plastic surgery is treating gingival recessions and other abnormalities impacting the mucogingival complex.^17^ Gingival recession is characterized by the exposure of the root surface due to an apical displacement of the free gingival margin beyond the cementoenamel junction (CEJ).^29^ Several potential risk factors, such as history of orthodontic therapy, plaque-induced inflammation, mechanical trauma, phenotype, and tooth malposition, may contribute to gingival recession.^16,34^ The overall prevalence of gingival recession among patients can reach up to 80%,^46^ with 40% of symptomatic cases showing a recession depth of > 5 mm.^52^ Gingival recession may undermine oral hygiene practice and esthetics, and predispose to root caries, dentin hypersensitivity, or further progression.^8,46^

Autogenous tissue grafts are considered the gold-standard approaches to treating gingival recession.^12^ However, these autogenous grafts require a second surgical site, typically the patient’s palate, which increases morbidity and postoperative discomfort.^57^ Minimally invasive approaches such as tunneling techniques and pedicle flaps, have emerged in recent years owing to their decreased tissue morbidity and increased patient acceptance.^35^ Therefore, in order to avoid harvesting from the patient’s palate, alternative graft sources and biomaterials are commonly utilized.^2,21^ In this context, platelet-rich fibrin (PRF), a second generation platelet concentrate,^11,51^ has emerged as an effective adjunct or standalone material for treating soft tissue recession, supported by extensive literature in cases with adequate keratinized tissue.^38,44^

The utilization of PRF as a tissue engineering scaffold has been explored in numerous studies,^23,50^ which have demonstrated that PRF is a superior scaffold in comparison with collagen in terms of cellular proliferation, angiogenesis, anti-microbial effects, and bone tissue engineering, leading to a more effective matrix for cell migration and cell adhesion.^9,18,45^ The fibrin matrix also serves as a barrier, mediating the expression of fibroblastic cells and growth factors as well as preventing epithelial down-growth, supporting their migration within the wound.^31,49^ The current evidence suggests that PRF membranes can be regarded as a three-dimensional mesh polymerized in a particular structure consisting of circulating stem cells, growth factors, leukocytes, and platelets, which permit optimal healing.^27,41^

Together with autologous platelet concentrates and biomaterials, emerging regenerative modalities such as exosome-based strategies have gained considerable attention for their potential to improve soft tissue healing and periodontal regeneration.^37^ Exosomes are extracellular vesicles ranging in size from 30 to 150 nm that facilitate cell-to-cell communication through the transfer of lipids, proteins, and microRNA.^26,43^ These signaling molecules play a critical role in enhancing several biological processes, including cell proliferation, differentiation, and angiogenesis, by acting on medicinal signaling cells.^5,32,36^ While exosomes are produced by all cells, their regenerative potential depends on the cellular source.^4,43^ Recent studies have demonstrated promising results utilizing exosomes derived from periodontal ligament stem cells, bone marrow stem cells, and dental pulp stem cells for tissue regeneration.^3,6,37^ Considering their regenerative potential, exosome-enriched PRF (Exos-PRF) represents a novel strategy to enhance GR treatment and promote periodontal healing.

While numerous in-vitro and animal models support the use of exosomes in regenerative dentistry,^15,33^ clinical evidence remains scarce. To date, the only reported clinical application in dentistry is a case report from our group, in which exosomes were utilized for ridge augmentation.^19^ Since solid PRF acts as a biological scaffold that can be enriched with bioactive molecules, the present case series aimed to assess the clinical efficacy of Exos-solid-PRF (utilizing exosomes of placental origin) in a minimally invasive vestibular incision technique for root coverage. The aim of this single-arm case series is to assess the clinical feasibility of utilizing exosomes in combination with platelet-rich fibrin as a regenerative treatment of gingival recessions. To the authors’ knowledge, this is the first human clinical report to investigate this novel strategy, offering preliminary evidence for the efficacy and safety of using Exos-solid-PRF for treating gingival recession.

## MATERIALS AND METHODS

### Study Outline

Prior to data collection, an Institutional Review Board (IRB) exemption was obtained for the retrospective chart analysis from Sterling IRB (ID:14131-NEstrin). The study included 27 consecutive patients treated between January 2023 and July 2024 in private practice setting by a single board-certified periodontist (Scott Froum DDS). Informed consent was provided prior to drawing blood to conduct the experiments detailed below.

All patients underwent the minimally invasive vestibular incision technique with the application of solid-PRF hydrated in exosomes (periosomes; proprietary exosomes prepared under Good Manufacturing Practices standards derived from placental tissue at NeoBiosis [Gainesville, FL, USA]) and liquid-PRF (details described below). Clinical parameters, including keratinized tissue width, gingival recession, and attached tissue levels, were recorded at baseline and reassessed at the 6-month follow-up.

### Exosome-enriched PRF preparation

**Fig 1:**
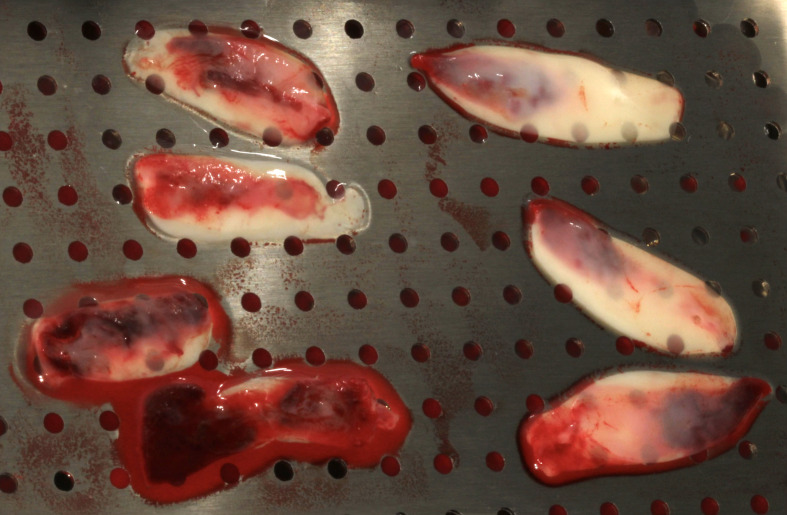
Solid-PRF clots, after compression, were hydrated in a solution containing exosomes mixed with liquid PRF.

For PRF preparation, four 10-ml tubes of the patient’s blood were drawn using an 18G needle. Two blue-top tubes were designated for liquid-PRF, while two red-top tubes were used for solid-PRF. The samples were centrifuged at 700 x g for eight minutes utilizing a horizontal centrifuge (Bio-PRF; Jupiter, FL, USA). After centrifugation, the solid-PRF clot was carefully separated from the red blood cell layer, ensuring the buffy coat remained intact before compression in the PRF-box to create flat PRF membranes. A total of 0.5 ml of liquid PRF was drawn into a 3-ml syringe and mixed with 0.5 ml of exosomes to hydrate the solid-PRF clots (Fig 1).

### Surgical Procedure 

Local anesthesia was administered at the treatment sites using

2% lidocaine HCl with 1:100,000 epinephrine.

Recipient sites were accessed utilizing the minimally invasive vestibular incision. For isolated recession defects, a minimal horizontal incision (2 – 3 mm) was made at the vestibular base, apical to the recession site. In cases of multiple adjacent defects, the incision was placed inter-radicularly. A transmucosal periosteal elevator was introduced through the vestibular hole for blunt dissection, creating a tunnel extending both laterally and coronally to mobilize the adjacent papillae and allow for coronal advancement of the mucogingival complex beyond the cementoenamel junction. Papilla release was done if necessary for proper flap advancement. Following the tunneling, Exos-solid-PRF clots were placed at the recession sites, after which the flap was coronally advanced and secured using Vicryl sutures in a sling fashion. The vestibular incision was closed with chromic gut in single interrupted fashion. A vestibular releasing incision was done in the event of a shallow vestibule and/or flap tension.^10^ Postoperative care included amoxicillin 500 mg thrice a day for three days, analgesics as required, and a homeopathic oral care recovery kit (StellaLife; Northbrook, IL, USA) for post-operative recovery.^20^ Brushing of the surgical area was avoided for two weeks. The patients were recalled for a postoperative evaluation at two weeks and a follow-up assessment at six months.

### Statistical Analysis

All statistical analyses were conducted using SPSS version 23.0 (IBM; Armonk, NY, USA). Continuous variables were expressed as mean ± standard deviation (SD). To evaluate changes in clinical parameters (i.e., attachment gain [AG], keratinized tissue [KT], recession depth [RD], and recession width [RW]) between baseline (pre-operative) and postoperative 6-month follow-up, a paired t-test was applied. Mean root coverage (%) was reported descriptively, as it represents a derived percentage value at a 6-month follow-up and was not subjected to statistical comparison. The level of statistical significance was set at p < 0.05. This case series has been reported in line with the PROCESS Guideline.^1^

## RESULTS

### Primary Features of Study Participants

A total of 27 patients were included in this study, comprising of 11 males (40.7%) and 16 females (59.3%), with a mean age of 37.8 years (range: 18 – 67 years). The total number of treated teeth was 125, with a median of 4 treated teeth per patient (range: 1 to 12 teeth). None of the patients reported a history of tobacco use. Among the cases, six involved isolated gingival recessions, while the remaining 21 cases presented multiple adjacent gingival recessions affecting 2 to 12 teeth per case. Regarding arch distribution, the majority of treated teeth were located in the maxilla (n=106; 84.8%), while 19 teeth (15.2%) were in the mandible. Maxillary-only treatments were performed in 22 patients (81.5%), mandibular-only treatments occurred in four patients (14.8%), and both arches were treated in one patient (3.7%). According to the Cairo classification system for recession, out of 125 teeth, 23 (18.4%) were RT1 and 102 (81.6%) were RT2. The most frequently treated teeth were in the anterior and premolar regions, predominantly in the esthetic zone (i.e., #15 to #25 in the maxilla and #33 to #43 in the mandible). The most treated single tooth was #23, appearing in six patients (22.2%), followed by #14 and #13, each appearing in five patients (18.5%) (Table 1).

**Table 1:** Primary features of the study participants

Patient	Gender	Number of teeth	Tooth number	Tooth Location
Case 1	M	6	13 to 23	Maxilla
Case 2	M	1	41	Mandible
Case 3	M	1	23	Maxilla
Case 4	M	3	23, 24, 25	Maxilla
Case 5	F	1	46	Mandible
Case 6	M	12	16 to 26	Maxilla
Case 7	F	4	15, 14, 24, 25	Maxilla
Case 8	F	6	11, 21, 33, 31, 41, 42	Maxilla, Mandible
Case 9	M	2	15, 14	Maxilla
Case 10	M	10	15 to 25	Maxilla
Case 11	F	8	14 to 24	Maxilla
Case 12	M	3	14, 13, 11	Maxilla
Case 13	F	6	33 to 43	Mandible
Case 14	F	4	15 to 12	Maxilla
Case 15	F	6	15 to 21	Maxilla
Case 16	F	8	14 to 24	Maxilla
Case 17	M	4	32 to 42	Mandible
Case 18	F	2	13, 23	Maxilla
Case 19	M	9	16 to 13, 22 to 26	Maxilla
Case 20	F	1	33	Mandible
Case 21	F	1	16	Maxilla
Case 22	F	2	31, 41	Mandible
Case 23	F	3	23 to 25	Maxilla
Case 24	F	2	14, 13	Maxilla
Case 25	M	12	16 to 26	Maxilla
Case 26	F	6	13 to 23	Maxilla
Case 27	F	2	14, 13	Maxilla


### Clinical Outcomes

**Fig 2:**
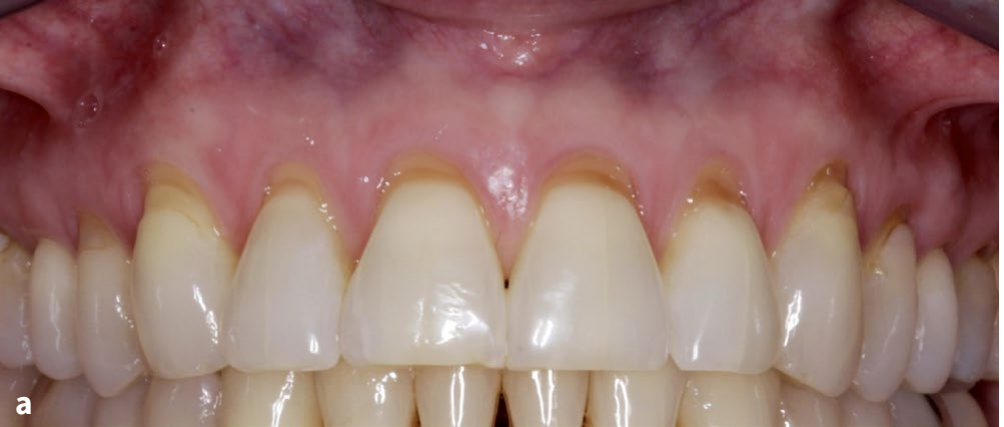

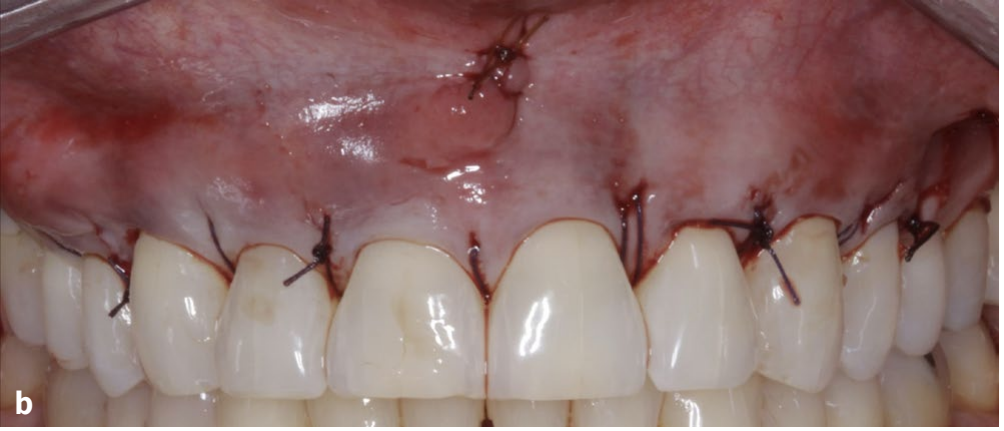

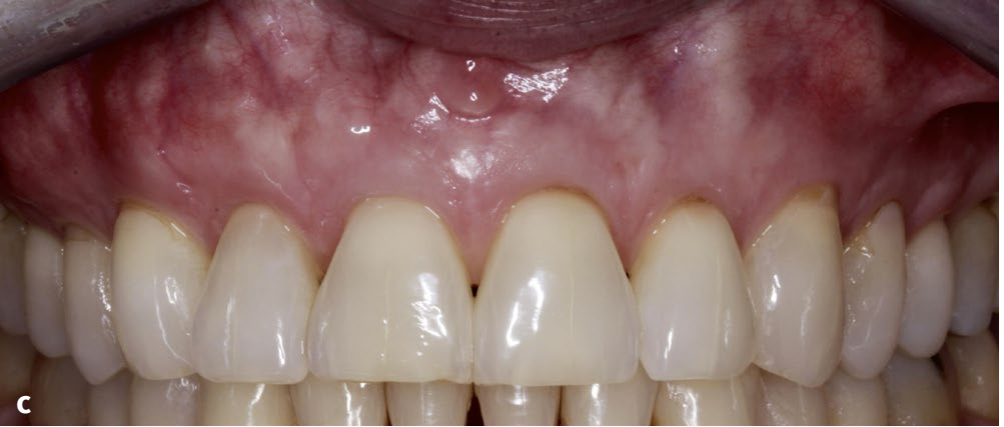
a) Pre-operative clinical photograph showing pronounced gingival recession in the maxillary region; b) Final suturing after the minimally invasive vestibular incision surgical technique, which was utilized to create access for placement of Exos-solid-PRF. c) Six-month post-operative follow-up.

**Fig 3:**
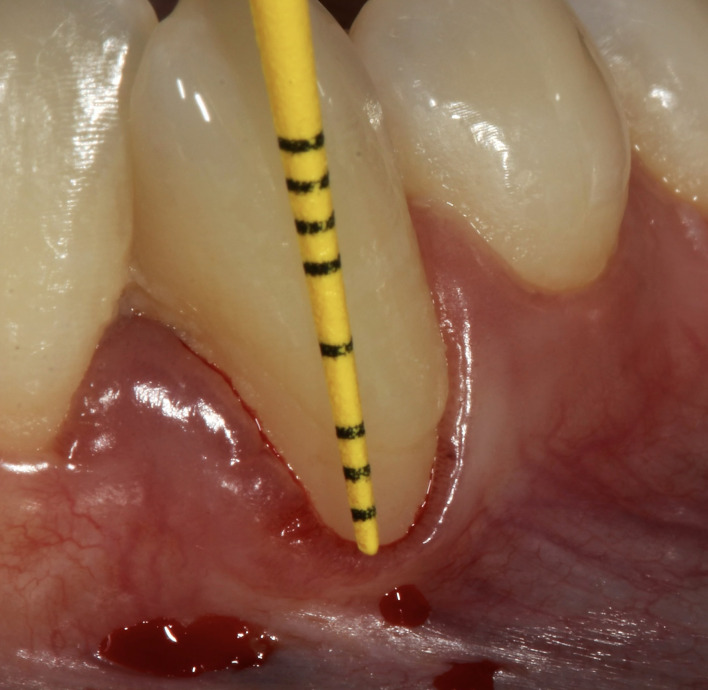

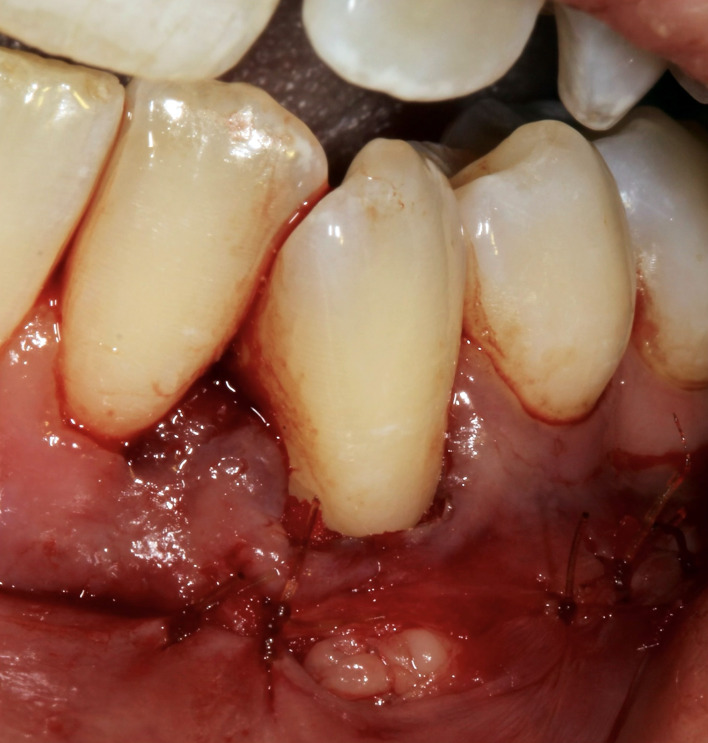

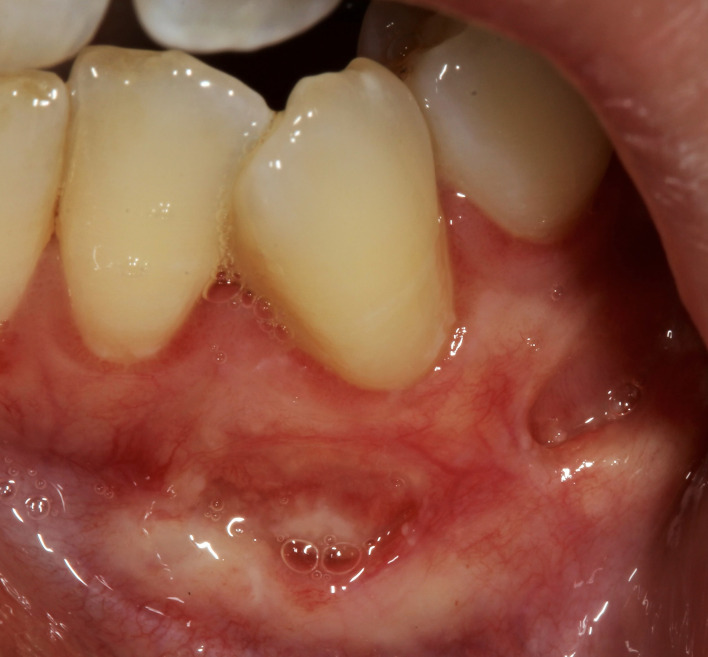

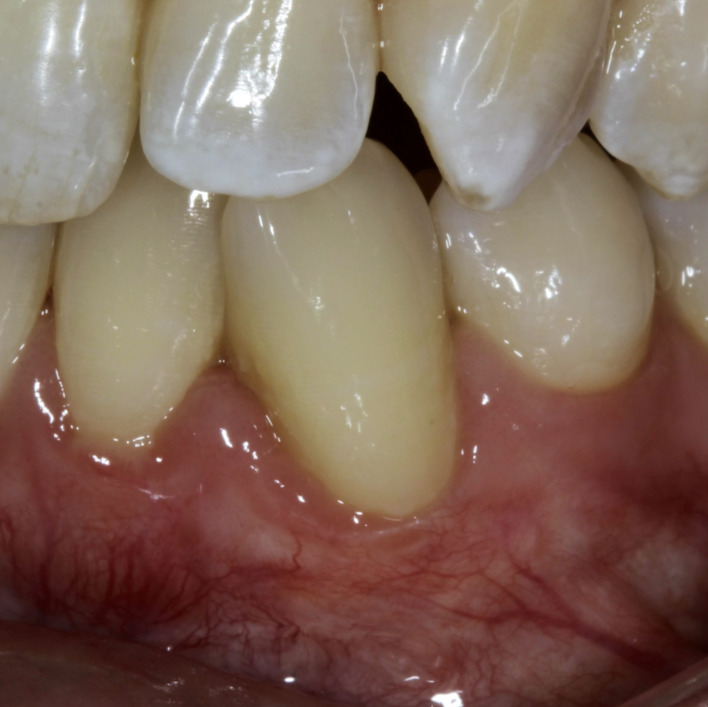
a) Clinical presentation of severe gingival recession at tooth #33; b) A minimally invasive vestibular incision surgical technique performed to create a tunneling approach, allowing placement of Exos-solid PRF on the buccal aspect of the recession defect (vestibular incision utilized to release tension); c) Six-week postoperative follow-up; and d) Six-month posteoprative follow-up demonstrating significant recession coverage.

**Fig 4:**
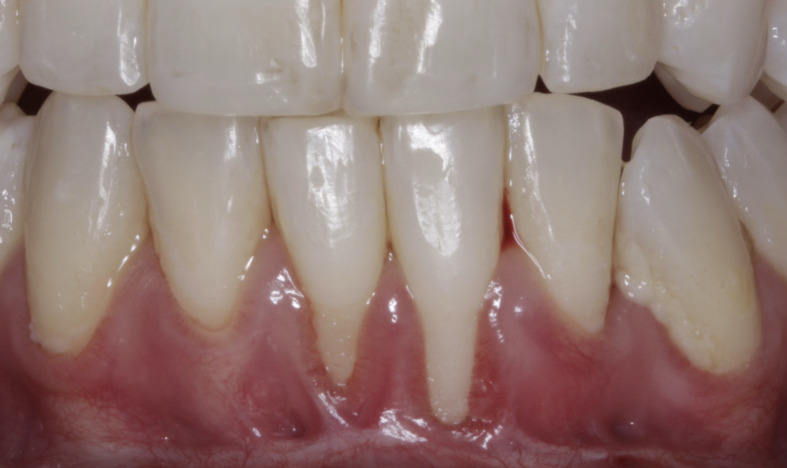

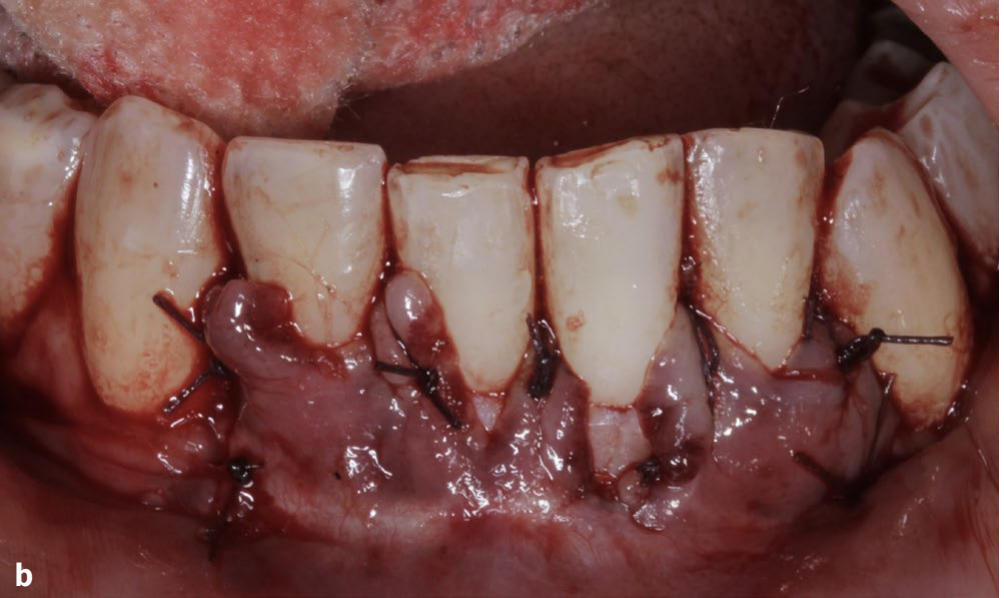

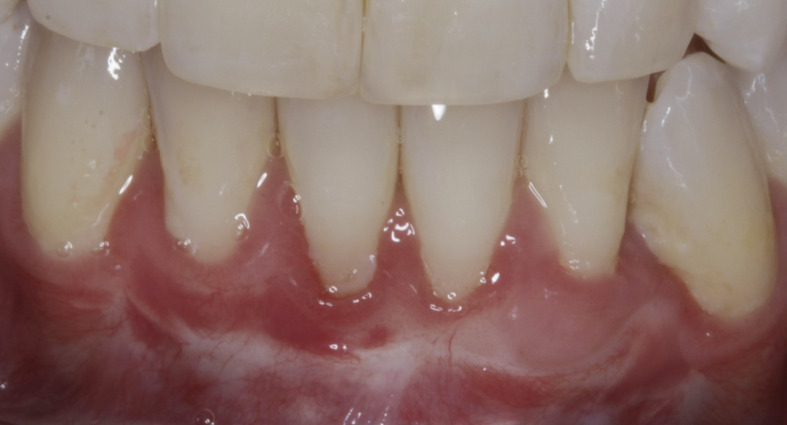
a) Pre-operative presentation showing severe gingival recession at teeth #41 and #31; b) Minimally invasive vestibular incision surgical technique performed with papilla release at sites #41 and #42), followed by placement of Exos-solid PRF on the buccal surface of the recession defect; and c) Six-month follow-up demonstrating marked improvement in recession coverage.

**Fig 5:**
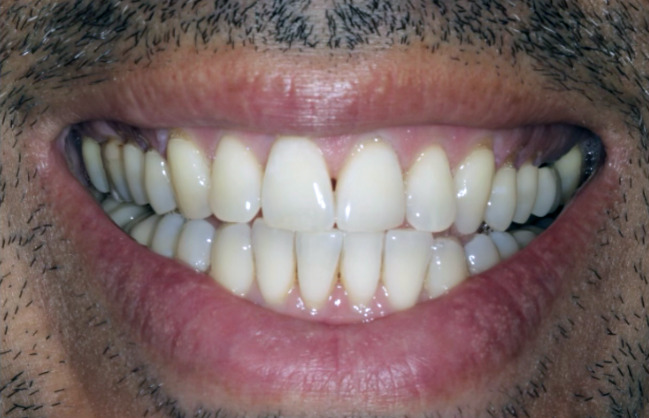

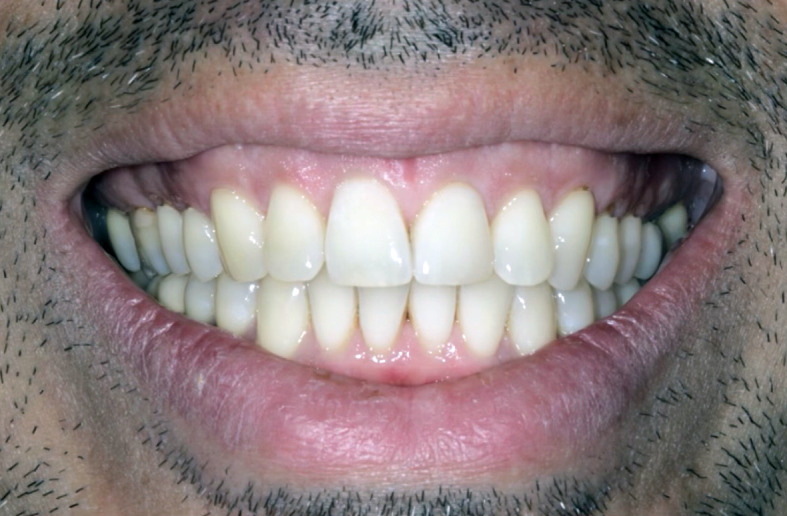
a) Pre-operative view; and b) Six-month postoperative results following treatment with a minimally invasive vestibular incision surgical technique utilizing Exos-solid-PRF. Note the considerable improvement in gingival coverage at sites #13, #21, and #23, including regeneration of the interdental papilla at sites #11/21.

**Fig 6:**
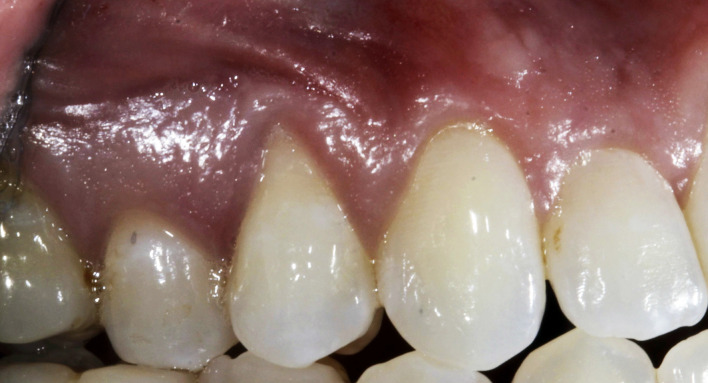

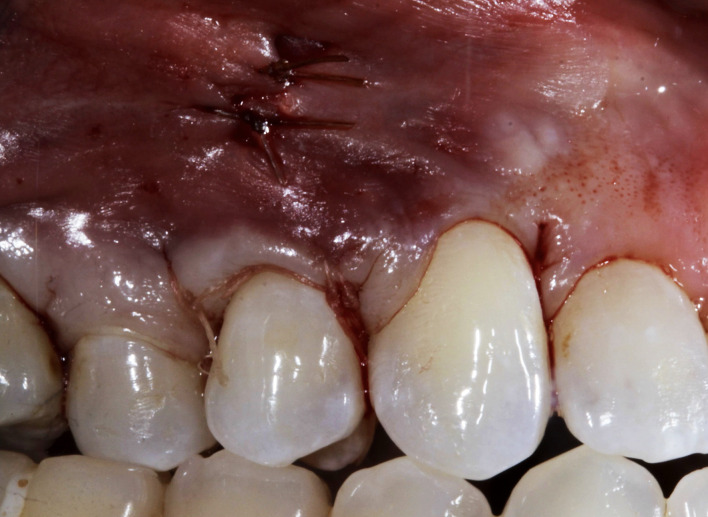

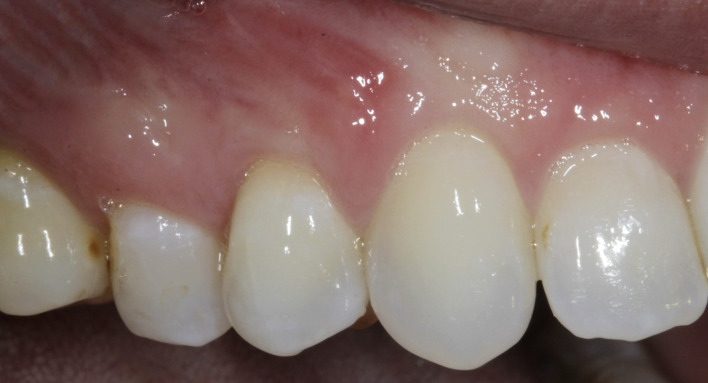
a) Pre-operative presentation showing severe recession at site #5 with a high frenum attachment; b) Minimally invasive vestibular incision surgical technique performed, and Exos-solid-PRF placed at the recession site; and c) Six-month follow-up showing significant resolution of the recession defect.

**Fig 7:**
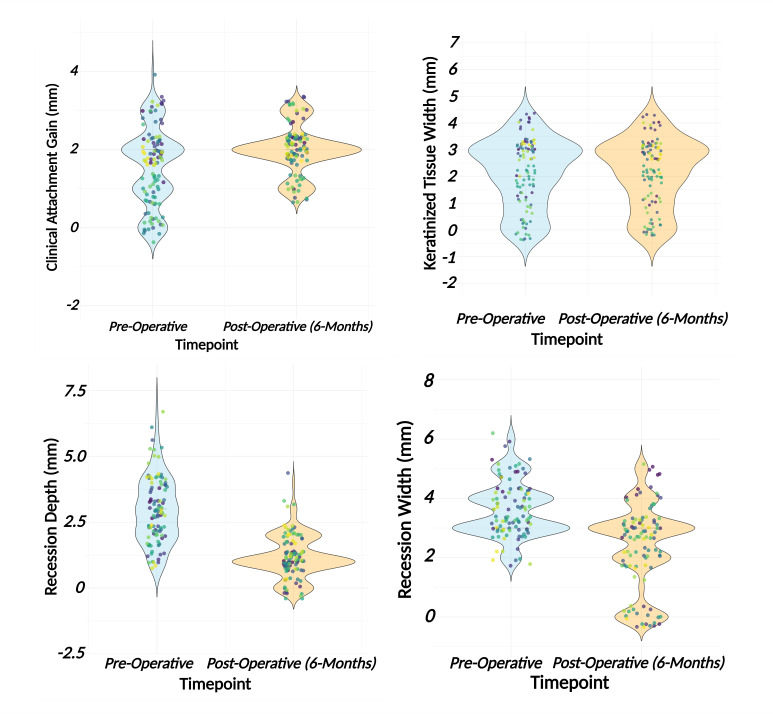
Violin plots comparing clinical outcomes at baseline (blue) and 6-month follow-up (pale orange) across 125 treated teeth receiving exosome-enriched solid platelet-rich fibrin via the minimally invasive vestibular incision surgical technique. (a) Attachment gains increased statistically significantly from baseline to 6 months; (b) keratinized tissue showed no change; (c) recession depth demonstrated a marked reduction; and (d) recession width also decreased statistically significantly postoperatively.

Figures 2 to 6 depict clinical cases presenting with gingival recession on at least one tooth as treated with a minimally invasive vestibular incision technique combined with Exos-solid-PRF. Table 2 presents the mean values of clinical outcomes associated with the use of Exos-solid-PRF at baseline and 6-month follow-up. The mean pre-operative AG was 1.53 ± 1.00 mm. At 6 months postoperatively, the mean AG value improved to 1.99 ± 0.59 mm (p < 0.001), reaching statistical significance with a mean change of +0.46 ± 0.84 mm. The mean pre-operative KT was 2.12 ± 1.25 mm, and the mean postoperative KT remained unchanged at 2.12 ± 1.25 mm, with no statistically significant change. The mean pre-operative RD was 3.02 ± 1.45 mm, which reduced to 1.08 ± 0.93 mm postoperatively (p < 0.001), reaching statistical significance with a mean change of -1.93 ± 1.05 mm. The mean pre-operative RW was 3.57 ±  0.94 mm, which decreased to 2.14 ± 1.48 mm postoperatively (p < 0.001), a statistically significant difference, with a mean change of -1.43 ± 1.43 mm. At the 6-month follow-up, the average percentage of root coverage across all 125 teeth was 68 ± 24%, with CRC achieved in 34 teeth. Of these, 14 were RT1 (Miller Class I or II), and 20 were RT2 (Miller Class-III). RT1 cases demonstrated an average root coverage of 86%, while RT2 cases showed a mean root coverage of 64%. Figure 7 depicts the tooth-level outcomes of Exos-solid-PRF on clinical AG, KT, RD, and RW.

**Table 2:** Mean values of clinical parameters assessed at baseline and 6-month follow-up

Clinical parameters	Pre-operative (baseline)	Post-operative (6 months)	Change	Significance* (p-value)
Attachment gain (mm)	1.53 ± 1.00	1.99 ± 0.59	0.46 ± 0.84	< 0.001
Keratinized tissue (mm)	2.12 ± 1.25	2.12 ± 1.25	0 ± 0	1.00
Recession depth (mm)	3.02 ± 1.45	1.08 ± 0.93	1.93 ± 1.05	< 0.001
Recession width (mm)	3.57 ± 0.94	2.14 ± 1.48	1.43 ± 1.43	< 0.001
Mean root coverage (%)	NA	68 ± 24	NA	NA
*Statistical significance set at p < 0.05.

The data generated in this study are available from the authors on request.

## DISCUSSION

This case series aimed to assess the clinical feasibility and efficacy of utilizing Exos-solid-PRF as a regenerative adjunct in the treatment of gingival recessions. All 27 patients demonstrated uneventful healing, with no adverse effects or complications, suggesting that this biologically enhanced approach is safe and well-tolerated. At the 6-month follow-up, the intervention yielded a statistically significant reduction of -1.93 ± 1.05 mm of recession, which is clinically significant and particularly notable considering the predominance of high-difficulty recession defects (RT2) included in the study cohort. While there was also a statistically significant gain of +0.46 ± 0.84 mm in attached gingiva, this may not be clinically significant/observable. Future larger scale clinical trials with longer-term follow-up are necessary to determine if this technique has a clinically significant effect on increasing attached gingiva.

Among the 125 treated teeth, CRC was achieved in 34 teeth (27.2%), of which 14 were RT1 (Miller Class I/II) and 20 were RT2 (Miller Class III). When stratified by classification, 60.9% of RT1 sites achieved CRC (with a mean root coverage of 86%), which is comparable to outcomes from previous studies using autogenous connective tissue grafts (CTGs) in combination with tunneling or CAF approaches, where root coverage values typically range between 85 and 95%.^7,25,58^ These comparable results are particularly important given that our approach utilized no autogenous donor tissue, which commonly contributes to increased patient morbidity and extended healing period. However, in the RT2 group – representing the majority of treated sites (81.6%) – only 19.6% achieved CRC, with an average root coverage of 64%. This is lower than the CRC rates (up to nearly 55%) reported in a systematic review assessing RT2 defects treated with autogenous grafts.^22^ The limited regenerative potential of RT2 defects, often owing to interproximal attachment loss and decreased vascularity, inherently complicates efforts for complete root coverage. Nevertheless, the ability of Exos-solid-PRF to obtain complete or partial coverage in these complex cases – without autogenous grafts – indicates a clinically meaningful biological contribution^7,25,58^ with further research needed to best optimize outcomes.

Research on PRF has consistently demonstrated its effectiveness in treating periodontal defects, such as gingival recession^14^ and intrabony/furcation defects.^39,40^ Recently, PRF was utilized for the treatment of recession type 2 (RT2) gingival recession, exhibiting significant esthetic improvements.^14^ Moreover, Jankovic et al^28^ conducted a randomized controlled trial assessing the application of a coronally advanced flap (CAF) with a PRF matrix. They found complete root coverage (CRC) in 80% of the CAF group and around 76% of cases within the PRF group. Furthermore, some case reports and case series indicate that a sub-pedicle PRF matrix, especially as an adjunct with a laterally displaced flap, can improve the predictability of pedicle flaps and achieve significant root coverage.^31^ Additionally, Gautam et al^24^ noticed an enhancement in clinical attachment levels at six-month follow-up, with recession coverage reaching up to 80%. In 2013, another study assessed a modified laterally sliding flap in combination with PRF for Class II gingival recession, showing 80% root coverage at the six-month follow-up.^56^ Moreover, Oncu et al^47^ suggested PRF as a viable alternative to subepithelial connective tissue grafts to treat multiple recessions.

Unlike traditional CTGs, which contribute both a structural and cellular component, the rationale of Exos-solid-PRF was to utilize PRF mainly as a bioactive scaffold and delivery mechanism for the exosomes, potentially improving cellular recruitment, angiogenesis, and extracellular matrix remodeling. The lack of significant gain in keratinized tissue, as noticed in this report, might be due to the absence of keratinized donor tissue inherent in CTG strategies.^30^ This finding aligns with the previous PRF reports, which found variable or limited impacts on KTW.^38^ In clinical settings where the augmentation of keratinized tissue is vital, the autogenous grafting method remains the superior option.

It is also imperative to contextualize our outcomes with the limitations of current evidence. A recent systematic review by Miron et al^38^ included 17 studies analyzing the adjunctive use of PRF in CAF procedures, all of which involved Miller Class I or II defects. They reported a modest but statistically significant improvement in root coverage (10-15%) with PRF; however, no study assessed higher-class recession defects including RT2.^38^ This gap in the current literature highlights the novelty and clinical relevance of the present study, which introduced Exos-solid-PRF as a potentially valuable option for Class III defects, where treatment predictability is inherently lower.

Further insights can be drawn from the review by Fernandez et al,^22^ which reported CRC rates in RT2 defects decreasing from 54.9% at 6 months to just 18.2% at > 12 months, underscoring the temporal instability of root coverage outcomes in such challenging cases. The current report observed that while short-term results in RT2 defects were promising, the durability of these outcomes remains unknown, which is a limitation of this study. Longer-term follow-up will be necessary to evaluate the stability and maintenance of clinical gains achieved with this novel approach.

From a biological aspect, the role of PRF as a scaffold able to entrap and deliver exosomes is mechanistically plausible but not yet empirically validated. The fibrin matrix theoretically facilitates the release of exosomes, permitting prolonged exposure of the site to regenerative signaling molecules, such as miRNAs, cytokines, and growth factors. Nonetheless, to date, no preclinical study has quantified the bioavailability, release kinetics, or entrapment efficiency of exosomes added to PRF matrices. However, several studies have been published supporting the use of PRF as a matrix for extended release of biomolecules such as antibiotics and Vitamin C.^39,42,55^ Further studies are required to elucidate the associations between the PRF matrices and exosomes specifically, including whether PRF improves the cellular uptake, stability, or regenerative potential of exosomes in situ.

An important limitation of this report is the absence of a comparison or control group which prevents clear attribution of clinical findings to the incorporation of exosomes. It remains unclear whether the observed improvements, especially in RT2 defects, are mainly due to the surgical approach, the exosomes, the PRF matrix, or a synergistic interaction among these components. Hence, future well-controlled randomized clinical studies comparing exosomes alone, PRF alone, and the combined Exos-PRF matrix are warranted to understand the additive and individual impacts of each component. Other limitations of this study include the lack of randomization which could introduce selection bias, and the lack of blinding in the outcome assessment, which could potentially introduce measurement bias. These issues should be addressed in future studies on this technique. Furthermore, while the vestibular incision and tunneling technique provides a minimally invasive alternative with decreased patient morbidity,^13^ its results are known to be technique-sensitive and might differ from the surgeon’s experience.^48^ Additionally, there were minor variations in technique, including papilla and/or vestibular release, which were applied depending on the clinical presentation of each case, a further limitation of this study. Standardization of the surgical approach and training requirements would be imperative for broader adoption and reproducibility of this approach. Future multicenter clinical trials with various operators are necessary to help minimize operator-related factors and improve reproducibility.

While our findings suggest potential added benefits from the addition of placenta-derived exosomes to PRF for root coverage procedures, cautious interpretation is advised due to the presence of several limitations, most notably the study design as a case-series lacking a control group. Despite these limitations, the current case series provides preliminary but valuable insights into the utilization of exosomes in periodontal plastic surgery. Given the increasing interest in exosome-based therapeutics, this study contributes early clinical evidence supporting their safety and feasibility in regenerative protocols for mucogingival defects. Importantly, this biologically driven strategy might be particularly appealing to patients who decline or are not candidates for autogenous grafting procedures.

## CONCLUSION

In this first human clinical study, exosomes were utilized for the first time for the treatment of gingival recession coverage procedures: Exos-solid-PRF was applied via a minimally invasive vestibular incision technique, exhibited promising short-term clinical efficacy in both RT1 and RT2 gingival recession. While the outcomes for RT1 defects are comparable to those of gold-standard CTG approaches, the modest improvements in RT2 defects underscore both the potential and the limitations of this novel method. Additional controlled studies, long-term follow-up, and mechanistic investigations are required to validate these outcomes and optimize the therapeutic application of exosomes in clinical periodontology.

## ACKNOWLEDGEMENT

Nima Farshidfar is a recipient of the 2024 Research Scholarship from the Osteology Foundation, Root, Switzerland. Conflict of interest: Richard J. Miron holds intellectual property on the production of PRF and is the founder of Miron Research and Development in Dentistry LLC. All other authors declare that they have no conflict of interest.
